# Psychological mechanisms of perceived technological dependence on generative artificial intelligence tools: perceived knowledge, trust, anxiety, and self-efficacy

**DOI:** 10.3389/fpsyg.2026.1928967

**Published:** 2026-10-23

**Authors:** Le Wei, Shu Zhang, Junghyun Heo

**Affiliations:** 1 Interaction Design Lab, Graduate School of Techno Design, Kookmin University, Seoul, Republic of Korea; 2 Anhui Institute of International Business, Hefei, Anhui, China; 3 Anhui Vocational College of Press and Publishing, Hefei, Anhui, China

**Keywords:** design education, generative AI tools, perceived AIGC knowledge, perceived technological dependence, trust, usage intention

## Abstract

Generative AI tools are increasingly used in design education, yet the relationship between students’ perceptions of these tools and their reported reliance on them remains unclear. This study analyzed cross-sectional survey responses from 526 undergraduate students in design-related disciplines using partial least squares structural equation modeling. In the specified model, perceived reliability, perceived usefulness, and perceived intelligence were positively associated with perceived AIGC knowledge, whereas perceived ease of use was not significantly associated with it. Perceived AIGC knowledge was positively associated with trust, self-efficacy, and usage intention and negatively associated with AIGC-related anxiety. Trust, AIGC-related anxiety, and self-efficacy each showed a significant indirect effect in the modeled relationship between perceived AIGC knowledge and usage intention. Usage intention was strongly associated with perceived technological dependence. These findings are consistent with the proposed framework, but the cross-sectional data do not establish the temporal sequence of the constructs or show that reported dependence reflects harmful overuse. The study extends technology acceptance research by examining perceived reliance in AIGC-supported creative work.

## 1 Introduction

Generative artificial intelligence has rapidly entered higher education and is reshaping how students access information, develop ideas, and complete learning tasks. Recent survey evidence suggests that the use of generative AI tools among university students has moved from occasional experimentation to routine academic practice. The Student Generative AI Survey 2025 reported that 92% of students had used AI tools in some form, a substantial increase from 66% in the previous year (Freeman, 2025). Similarly, the Global AI Student Survey 2024 found that 86% of students used AI in their studies, with 54% using it at least weekly (Digital Education Council, 2024). Together, these developments indicate that generative AI is no longer a peripheral technology in higher education but an increasingly embedded resource in students’ learning practices (Chan and Hu, 2023; Crompton and Burke, 2023; Kasneci et al., 2023; Zawacki-Richter et al., 2019).

This transformation is particularly salient in design-related disciplines. In this study, design-related disciplines refer to fields such as visual communication, graphic and brand design, industrial and product design, interaction/UI/UX design, and environmental, interior, and landscape design. These fields commonly require students to engage in creative ideation, visual exploration, sketch development, style referencing, and iterative refinement. Compared with conventional digital design tools, AI-generated content (AIGC) tools—broadly referring to generative AI tools that produce textual, visual, or multimodal content—can support students in generating alternatives, exploring styles, accelerating early-stage design development, and refining creative directions (Jiao and Cao, 2024; Verganti et al., 2020). Prior research has shown that students’ acceptance of AIGC-assisted design courses is shaped not only by technological factors but also by instructional factors, course design, and learning resources (Jiang et al., 2024). Similarly, recent work on art and design students has examined how AIGC-assisted learning and design creation are influenced by perceived usefulness, perceived ease of use, attitudes, and behavioral intention within an extended TAM framework (Zhu et al., 2025). These studies provide an important foundation for understanding students’ acceptance of AIGC in design education.

However, the increasing integration of AIGC into design learning raises concerns that go beyond initial technology acceptance. In design education, AIGC does not merely help students retrieve information or automate routine tasks; it may intervene directly in idea generation, aesthetic judgment, visual decision-making, and creative authorship. Students may benefit from enhanced efficiency and inspiration, but they may also struggle to evaluate the reliability, originality, and appropriateness of AI-generated outputs. Research on designer trust in AIGC has shown that trust plays a critical role in shaping designers’ behavioral intentions, particularly when uncertainty and perceived risk are involved (Wang and Chen, 2024). More broadly, systematic review evidence suggests that students’ over-reliance on AI dialog systems may weaken critical cognitive capabilities, including decision-making, critical thinking, and analytical reasoning, especially when users accept AI-generated outputs without sufficient evaluation (Zhai et al., 2024). These concerns are especially relevant to design education, where students are expected not only to produce visually compelling outcomes but also to develop independent creative judgment.

Existing research on generative AI in higher education has largely focused on adoption, acceptance, and behavioral intention. The Technology Acceptance Model (TAM) has been widely used to explain users’ acceptance of information technologies through perceived usefulness and perceived ease of use (Davis, 1989; Venkatesh and Davis, 2000). Recent studies have extended TAM or related models to examine GenAI and AIGC acceptance in educational contexts by incorporating factors such as trust, anxiety, self-efficacy, social influence, instructional support, and perceived usefulness (Jiang et al., 2024; Zhu et al., 2025). Although these studies have advanced understanding of why students intend to use generative AI tools, they tend to remain centered on the adoption stage. Less attention has been paid to what happens after adoption, particularly whether intention-driven use may become associated with perceived technological dependence.

A further question concerns what students believe they know about the AIGC tools they use. In design work, students must decide whether generated material suits a task and how it might be incorporated into their own work. Research on GenAI literacy places such judgments within a broader set of competencies that includes understanding, applying, and evaluating generative AI (Park, 2025). The present study does not assess those competencies through a knowledge test or a design task. Instead, it examines students’ reported understanding of AIGC tools and whether that perception is associated with their psychological responses and intention to use the tools.

Against this background, this study develops and tests an extended TAM framework to examine how design students’ AIGC-related technological perceptions and perceived knowledge are associated with psychological responses, usage intention, and perceived technological dependence. Rather than treating AIGC use merely as a question of technology acceptance, this study approaches AIGC-supported design creation as a cognitive process in which students interact with AI-generated outputs, evaluate uncertainty, form judgments about whether to trust AIGC outputs, regulate anxiety, and decide whether and how to integrate generated materials into creative work. This view is consistent with recent work on GenAI literacy and AI over-reliance, which suggests that effective use of generative AI requires not only access to tools but also the ability to evaluate, incorporate, and critically reflect on AI-generated outputs (Park, 2025; Zhai et al., 2024).

Specifically, the model examines how perceived reliability, perceived ease of use, perceived usefulness, and perceived intelligence contribute to students’ perceived AIGC knowledge. It then investigates how perceived AIGC knowledge is associated with trust, AIGC-related anxiety, self-efficacy, and usage intention, and how usage intention relates to perceived technological dependence. By doing so, the study shifts the analytical focus from whether students accept AIGC tools to how perceived knowledge and psychological responses are associated with emerging reliance on AI-supported creative resources.

Accordingly, this study addresses the following research questions:

*RQ1*: What technological perceptions contribute to design students’ perceived AIGC knowledge in AIGC-supported creative tasks?

*RQ2*: To what extent do trust, AIGC-related anxiety, and self-efficacy explain the association between perceived AIGC knowledge and usage intention?

*RQ3*: How does usage intention relate to perceived technological dependence on AIGC tools?

To answer these questions, survey data were collected from 526 students in design-related disciplines and analyzed using partial least squares structural equation modeling (PLS-SEM). This methodological approach is appropriate for examining complex prediction-oriented models involving multiple endogenous constructs and indirect effects in emerging technology contexts.

This study makes three contributions to the literature. First, it extends technology acceptance research by moving beyond usage intention to examine perceived technological dependence as a post-adoption outcome of AIGC-supported creative work. Second, it conceptualizes perceived AIGC knowledge as a form of cognitive readiness that connects technological perceptions with trust, anxiety, self-efficacy, and intention. Third, it identifies distinct statistical indirect associations between Perceived AIGC Knowledge and Usage Intention through Trust, AIGC-related Anxiety, and Self-efficacy. Together, these analyses describe how perceived understanding, psychological responses, intention, and reported reliance are associated in AIGC-supported design work.

## 2 Literature review and hypothesis development

### 2.1 AIGC-supported design creation

AI-generated content (AIGC) tools refer to generative AI systems that produce textual, visual, or multimodal content in response to user prompts. In this study, the term encompasses generative AI applications used in design creation, including text-based, image-generating, and multimodal tools; it is not limited to text-to-image systems. Participants were eligible based on their reported experience using AIGC tools for design tasks, so the findings concern AIGC-supported design work rather than a particular type of tool or platform. In design education, their relevance lies not only in technical efficiency but also in their proximity to the creative process itself. Design students often work through uncertain and iterative processes: they search for ideas, test visual directions, compare stylistic references, develop sketches, and refine proposals through repeated adjustments. AIGC tools can enter this process at an early stage by providing alternative concepts, visual prompts, composition references, and stylistic possibilities. In this respect, they differ from conventional digital design tools, which mainly support execution after a design direction has already been formed. This role is consistent with broader discussions of AI-supported design and innovation, where AI systems are increasingly understood as tools that can expand ideation, support exploration, and reshape creative workflows (Jiao and Cao, 2024; Verganti et al., 2020).

Recent research has begun to examine how AI and generative AI are being incorporated into higher education, highlighting both their instructional potential and the challenges they pose for teaching, assessment, and student learning (Crompton and Burke, 2023; Kasneci et al., 2023; Zawacki-Richter et al., 2019). In the specific context of design education, Jiang et al. (2024) showed that students’ acceptance of AIGC-assisted design courses is influenced by both technological and instructional factors, indicating that AIGC use in design education cannot be reduced to tool performance alone. Course structure, learning resources, and pedagogical support also shape how students evaluate and engage with AIGC-assisted learning. In a related study, Zhu et al. (2025) examined art and design students’ acceptance of generative AI-assisted learning and design creation through an extended TAM framework, highlighting the continuing relevance of perceived usefulness, perceived ease of use, and behavioral intention in this context.

These studies provide a necessary starting point, but they also reveal a limitation in the current literature. Much of the existing work still asks whether students accept AIGC tools or intend to use them. This question is important, but it does not fully capture what is at stake in design education. AIGC tools are not only used to retrieve information or complete routine learning tasks. They may also shape how students generate ideas, judge visual quality, and make creative decisions. For this reason, research on AIGC use in design education should examine students’ perceived knowledge, trust, self-efficacy, anxiety, and reported reliance on AI-supported creative resources alongside technology acceptance.

### 2.2 From technology adoption to perceived technological dependence

The Technology Acceptance Model (TAM) remains one of the most widely used frameworks for explaining technology acceptance. Its core assumption is that users’ behavioral intention is shaped primarily by perceived usefulness and perceived ease of use (Davis, 1989). Later extensions of TAM showed that users’ acceptance of technology can also be influenced by broader social, cognitive, and contextual factors (Venkatesh and Davis, 2000). This theoretical development is also reflected in the Unified Theory of Acceptance and Use of Technology and its later extension, which further emphasized the role of performance expectancy, effort expectancy, facilitating conditions, social influence, and user context in technology acceptance (Venkatesh et al., 2003; Venkatesh et al., 2012). This flexibility explains why TAM and its extensions have been repeatedly adopted in educational technology studies, including research on e-learning, mobile learning, learning management systems, and, more recently, generative AI tools.

Recent information systems research has broadened how quality is considered in digital learning. Salam and Farooq (2026) proposed a hierarchy of quality dimensions and an updated IS success model that incorporates social quality in computer-supported collaborative learning. In AI adoption research, the revised artificial intelligence device use acceptance (RAIDUA) model examines usefulness expectancy, emotional responses, and privacy concerns in the acceptance of AI-enabled devices (Salam et al., 2026). These frameworks provide context, but the present study measured students’ current perceptions of AIGC tools rather than their prior expectations or a hierarchy of quality dimensions. Social quality and privacy concerns were not assessed.

In the case of AIGC, however, the original TAM framework is useful but incomplete. Generative AI tools do more than provide access to resources or support task execution. They generate content, offer suggestions, simulate interaction, and produce outputs that may be treated by students as creative or cognitive support. As a result, students’ acceptance of AIGC tools may involve not only usefulness and ease of use, but also uncertainty, trust, perceived control, and anxiety. This is why recent studies on AIGC and GenAI adoption have increasingly extended TAM by incorporating factors such as trust, anxiety, self-efficacy, instructional support, and perceived system capability (Jiang et al., 2024; Zhu et al., 2025).

A further issue is that acceptance does not necessarily end with intention. In information systems research, continuance intention has been widely used to explain users’ willingness to keep using a technology after initial adoption (Bhattacherjee, 2001). Habit research further suggests that repeated technology use may develop into automatic behavioral patterns that limit the predictive power of intention (Limayem et al., 2007; Verplanken and Orbell, 2003). In AIGC-supported learning and creation, however, the more pressing question is not only whether students intend to continue using AIGC or whether their use becomes habitual, but whether they begin to perceive AIGC tools as necessary for completing tasks efficiently or producing satisfactory creative outcomes. This issue has become increasingly visible in education and automation research, where scholars have raised concerns about reliance and over-reliance on AI-supported systems (Klingbeil et al., 2024; Parasuraman and Riley, 1997; Zhai et al., 2024).

In this study, perceived technological dependence refers to students’ self-reported reliance on AIGC tools in their current design work. It encompasses the incorporation of these tools into routine tasks, a preference for using them in design creation, and the expectation that working without them would reduce efficiency. We use “dependence” in this limited, functional sense. The construct does not imply compulsive use, impaired functioning, or objectively demonstrated inability to design without AI support.

This definition places perceived technological dependence alongside, but not entirely apart from, other post-adoption constructs. Usage intention concerns students’ plans to use AIGC tools in future work, whereas habit concerns the automaticity of repeated behavior (Bhattacherjee, 2001; Limayem et al., 2007; Verplanken and Orbell, 2003). Our measure includes an item on habitual reliance, so some conceptual proximity to habit is expected. Its distinguishing emphasis is students’ reported incorporation of AIGC into their design process and their anticipated difficulty in maintaining efficiency without it. The findings are therefore interpreted as evidence about perceived functional reliance, not actual or harmful over-reliance (Parasuraman and Riley, 1997; Zhai et al., 2024).

### 2.3 Perceived AIGC knowledge as cognitive readiness

One limitation of many TAM-based studies is that they implicitly assume that users are able to evaluate the technology they are asked to accept. This assumption is less stable in the context of AIGC. Generative AI tools often appear easy to access and operate, but responsible and meaningful use requires more than knowing how to enter a prompt. Students need to understand what these tools can and cannot do, how generated outputs are shaped by prompts and training data, why outputs may be inaccurate or visually misleading, and how AI-generated content should be evaluated before being incorporated into disciplinary work.

In this study, Perceived AIGC Knowledge refers to students’ self-reported understanding of AIGC tools in design creation, including how to use them and when they are suitable for a task. This focus is informed by research on AI and GenAI literacy (Laupichler et al., 2022; Long and Magerko, 2020; Ng et al., 2021; Park, 2025), but it is narrower than literacy as a demonstrated ability. The questionnaire records students’ judgments about their own understanding; it does not establish whether they can accurately explain how a system works or assess the quality of its outputs.

These judgments may be related to how students view the tools they use. Students who perceive an AIGC tool as reliable, useful, or intelligent may also report a better understanding of it. Their reported understanding may, in turn, be associated with trust, AIGC-related anxiety, self-efficacy, and usage intention. The model examines these relationships in the context of design creation, while leaving open whether students’ perceived knowledge corresponds to their actual competence.

### 2.4 Theoretical positioning: psychological responses and perceived technological dependence

Taken together, prior research has established that AIGC tools are becoming increasingly relevant to design-related creative work and that users’ acceptance of such tools can be explained, at least partly, through technology acceptance perspectives (Davis, 1989; Venkatesh and Davis, 2000). However, AIGC use in creative tasks cannot be understood only as a matter of adoption. It also involves how users interpret AI-generated outputs, evaluate uncertainty, decide whether to trust them, regulate anxiety, and maintain a sense of agency when working with intelligent tools (Lee and See, 2004; Park, 2025; Zhai et al., 2024).

This study is positioned at the intersection of technology acceptance research and psychological research on human-AI interaction. In AIGC-supported design creation, students do not simply operate a tool to complete predefined tasks. They engage with generated suggestions, visual alternatives, stylistic references, and design possibilities that may influence their creative judgment and decision-making. The present study therefore examines students’ reported Trust, AIGC-related Anxiety, Self-efficacy, Usage Intention, and Perceived Technological Dependence alongside their perceptions of AIGC tools. It treats Perceived AIGC Knowledge as their subjective understanding of tool functions, usage procedures, application contexts, and limitations, without assuming that this understanding demonstrates an ability to evaluate generated outputs.

The present study extends TAM by examining associations beyond Usage Intention. It places technological perceptions, Perceived AIGC Knowledge, psychological responses, Usage Intention, and Perceived Technological Dependence in a theoretically ordered model. This ordering follows the proposed framework; it does not represent an observed sequence of changes in the students. As discussed in Section 2.2, Perceived Technological Dependence concerns reported functional reliance on AIGC tools and has some conceptual proximity to continuance intention and habit (Bhattacherjee, 2001; Limayem et al., 2007; Verplanken and Orbell, 2003).

## 3 Research model and hypothesis development

The research model specifies hypothesized associations among students’ technological perceptions, Perceived AIGC Knowledge, psychological responses, Usage Intention, and Perceived Technological Dependence. Drawing on TAM and research on Trust, AIGC-related Anxiety, and Self-efficacy, it includes Perceived Reliability, Perceived Ease of Use, Perceived Usefulness, and Perceived Intelligence as proposed correlates of Perceived AIGC Knowledge. The model then examines the associations of Perceived AIGC Knowledge with Trust, AIGC-related Anxiety, Self-efficacy, and Usage Intention, followed by the association between Usage Intention and Perceived Technological Dependence. The arrows in Figure 1 indicate the hypothesized directions of these relationships, not a temporal sequence established by the survey.

**Figure 1:**
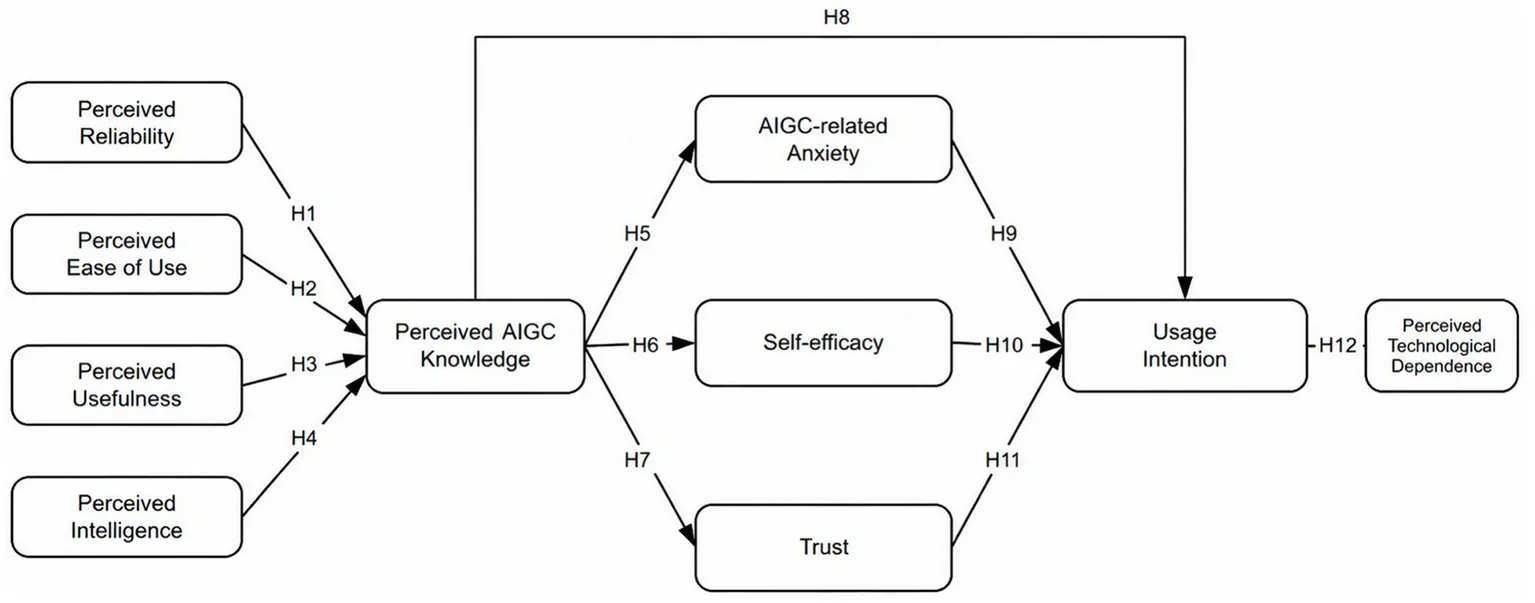
Proposed research model of AIGC-supported creative cognition and perceived technological dependence. The four technological perceptions are antecedents; AIGC-related Anxiety, Self-efficacy, and Trust are psychological mediators; Usage Intention and Perceived Technological Dependence are the modeled outcomes. The arrows in figure indicate hypothesized associations; they do not show when changes in the constructs occurred.

### 3.1 Technological perceptions and perceived AIGC knowledge

#### 3.1.1 Perceived reliability → perceived AIGC knowledge

Perceived reliability refers to students’ judgment that an AIGC tool can provide stable, consistent, and dependable support during design creation. In information systems research, system reliability and trust-related beliefs have been shown to shape users’ willingness to engage with digital technologies and rely on system outputs (DeLone and McLean, 2003; Gefen et al., 2003; McKnight et al., 2002). Similarly, human–automation research suggests that perceived reliability is closely associated with users’ willingness to rely on automated systems and treat system outputs as meaningful information (Hoff and Bashir, 2015; Lee and See, 2004). In AIGC-supported design creation, reliability is particularly important because students must decide whether generated outputs are worth further exploration, revision, or incorporation into their own work. If students perceive an AIGC tool as unreliable, they may interact with it only superficially or dismiss its outputs as unstable. By contrast, students who perceive the tool as dependable may also report a stronger understanding of its capabilities and boundaries. Therefore, the following hypothesis is proposed:

*H1*: Perceived reliability is positively associated with students’ perceived AIGC knowledge.

Perceived ease of use refers to the extent to which students believe that using AIGC tools requires little effort. It is an established factor in technology acceptance research (Davis, 1989; Venkatesh, 2000). In design creation, an interface that is easier to use may give students more opportunities to try different functions and become familiar with the tool. Whether this perceived ease of use is also associated with students’ reported understanding of AIGC tools is less clear. H2 examines that relationship.

*H2*: Perceived ease of use is positively associated with students’ Perceived AIGC knowledge.

Perceived usefulness reflects students’ belief that AIGC tools can improve their design learning or creative performance. In TAM and its extensions, perceived usefulness has consistently been treated as a key determinant of users’ intention to engage with a technology (Davis, 1989; Venkatesh and Davis, 2000). In design creation, usefulness may be experienced when AIGC tools help students generate alternatives, speed up early ideation, improve visual exploration, or provide references for further development. Students who perceive AIGC tools as useful for design tasks may also report a clearer understanding of how the tools work and where their outputs can be used.

*H3*: Perceived usefulness is positively associated with students’ Perceived AIGC knowledge.

Perceived intelligence refers to students’ perception that an AIGC tool can understand prompts, respond meaningfully, and generate outputs that appear contextually appropriate. Research on intelligent agents suggests that perceived intelligence can influence users’ adoption of AI-enabled systems because intelligent behavior makes the system appear more capable and responsive (Moussawi and Koufaris, 2021). In design education, perceived intelligence may be especially important because students often expect AIGC tools to interpret creative prompts, respond to stylistic intentions, and support open-ended exploration. Students who perceive an AIGC tool as intelligent may also report a clearer understanding of how prompts relate to outputs and where the tool may be useful in design creation.

*H4*: Perceived intelligence is positively associated with students’ Perceived AIGC knowledge.

### 3.2 Perceived AIGC knowledge, trust, AIGC-related anxiety, and self-efficacy

Perceived AIGC Knowledge refers to students’ perceived understanding of AIGC functions, usage procedures, appropriate application contexts, and limitations in design creation. As discussed in the previous section, this perceived understanding can be considered a form of cognitive readiness. Students who report greater Perceived AIGC Knowledge may also report less uncertainty when evaluating generated outputs for design tasks. AIGC-related anxiety refers to feelings of tension, uncertainty, or lack of control when using AIGC tools in these tasks.

*H5*: Perceived AIGC knowledge is negatively associated with AIGC-related anxiety.

Perceived AIGC Knowledge may also be associated with self-efficacy. Self-efficacy refers to an individual’s belief in their ability to perform a specific task successfully (Bandura, 1997). Prior research has linked computer self-efficacy to confidence in technology-enabled tasks (Compeau and Higgins, 1995). In AIGC-supported design creation, students who report greater understanding of the tools’ functions, procedures, and limitations may also report feeling more capable of refining prompts, selecting useful outputs, and integrating AI-generated materials into their own work. Therefore, Perceived AIGC Knowledge is expected to be positively associated with self-efficacy.

*H6*: Perceived AIGC knowledge is positively associated with self-efficacy.

In this study, Trust refers to students’ confidence in AIGC tools and their willingness to rely on AIGC-generated suggestions in design tasks. Trust in automated or AI systems is closely related to users’ perceptions of capability, reliability, predictability, and appropriate reliance (Glikson and Woolley, 2020; Hoff and Bashir, 2015; Lee and See, 2004; Mayer et al., 1995; McKnight et al., 2002). In design creation, students may decide whether to use, revise, or reject generated outputs. Students with stronger Perceived AIGC Knowledge may also report greater Trust in the tool; the present study cannot determine whether that Trust corresponds to actual tool reliability. Thus, Perceived AIGC Knowledge is expected to be positively associated with Trust.

*H7*: Perceived AIGC knowledge is positively associated with trust.

Beyond its associations with psychological responses, Perceived AIGC Knowledge may also be directly associated with Usage Intention. Students who report a clearer understanding of AIGC tools and their applications in design tasks may also express a stronger intention to use them in future creative work. This expected association is relevant to AIGC-supported design, where students need to judge whether generated outputs suit a task before deciding to use them.

*H8*: Perceived AIGC knowledge is positively associated with usage intention.

### 3.3 Psychological mechanisms and usage intention

AIGC-related anxiety may discourage students from using AIGC tools. Prior technology acceptance research has shown that anxiety and negative affect can reduce users’ willingness to engage with new technologies (Venkatesh, 2000). In the AIGC context, anxiety may arise from uncertainty about output quality, fear of losing creative control, concern about originality, or doubt about one’s ability to use the tool properly. When students feel tense or uncertain during AIGC-supported creation, they may avoid using the tool or limit its role in their design process. Therefore, AIGC-related anxiety is expected to be negatively associated with usage intention.

*H9*: AIGC-related anxiety is negatively associated with usage intention.

Self-efficacy is expected to have the opposite effect. Students who believe they can use AIGC tools effectively are more likely to experiment with prompts, evaluate outputs, and incorporate useful results into their design work. In technology acceptance research, self-efficacy has been widely linked to users’ willingness to engage with digital systems because it strengthens perceived control and reduces barriers to action (Compeau and Higgins, 1995; Venkatesh, 2000). In AIGC-supported design creation, self-efficacy may help students treat the tool as a controllable resource rather than an uncertain external force. Thus, students with higher self-efficacy are expected to show stronger usage intention.

*H10*: Self-efficacy is positively associated with usage intention.

Trust is also expected to encourage usage intention. When students believe that AIGC tools can provide useful, stable, and relevant support, they may be more willing to integrate these tools into their creative workflow. Trust is particularly important when users cannot fully inspect or predict system operations, as is often the case with AI systems (Lee and See, 2004). In design tasks, trust can lower perceived risk and make students more comfortable using AI-generated outputs as references, prompts, or starting points for further refinement. Accordingly, trust is expected to be positively associated with usage intention.

*H11*: Trust is positively associated with usage intention.

### 3.4 Usage intention and perceived technological dependence

Usage intention describes students’ stated willingness to use AIGC tools in future design work (Ajzen, 1991; Davis, 1989; Venkatesh et al., 2003; Venkatesh and Davis, 2000). Perceived technological dependence, as defined here, concerns the place these tools already occupy in students’ reported design practices and the efficiency they expect to lose without them. These constructs may be related, but willingness to use a tool and a sense of relying on it address different questions.

Research on technology continuance, habit, and automation reliance provides a basis for examining reliance among people who have already used a tool (Bhattacherjee, 2001; Lee and See, 2004; Limayem et al., 2007; Parasuraman and Riley, 1997). Although TAM commonly treats intention as an outcome, we place Perceived Technological Dependence after Usage Intention to examine whether students who intend to use AIGC more also report greater reliance on it in their current design work. Here, “post-adoption” refers to reliance reported by students with prior AIGC experience; it does not indicate a sequence observed in the data. Both variables were measured at the same time, so the reverse ordering remains possible. Accordingly, we propose the following hypothesis:

*H12*: Usage intention is positively associated with perceived technological dependence on AIGC tools.

The complete research model is summarized in Figure 1.

## 4 Materials and methods

### 4.1 Participants and data collection

Between June and August 2025, the questionnaire link was shared in course-related and student group chats to recruit undergraduate students in design-related disciplines in China. The questionnaire introduction explained the academic purpose of the study and stated that participation was voluntary. Respondents were eligible if they reported prior use of AIGC tools in design creation. Of the 537 submitted questionnaires, 11 were excluded because the respondents reported no such experience, leaving 526 for analysis. No personally identifiable or sensitive information was collected. Prior formal training in generative AI was not recorded in the questionnaire.

Table 1 describes the 526 eligible respondents. The sample included 270 male students (51.3%) and 256 female students (48.7%). There were 158 first-year students (30.0%), 150 second-year students (28.5%), 119 third-year students (22.6%), and 99 fourth-year students (18.8%). Regarding AIGC use experience, 204 reported occasional use (38.8%), 182 reported frequent use (34.6%), and 140 described themselves as highly proficient users (26.6%). Respondents came from visual communication/graphic/brand design, industrial/product design, product/UI/UX design, and environmental/interior/landscape design.

**Table 1:** Demographic characteristics of the respondents (*N* = 526).

Characteristic	Category	Frequency	Percentage (%)
Gender	Male	270	51.3
	Female	256	48.7
Academic year	First year	158	30
	Second year	150	28.5
	Third year	119	22.6
	Fourth year	99	18.8
AIGC use experience	Never used	0	0
	Occasional use	204	38.8
	Frequent use	182	34.6
	Highly proficient	140	26.6
Major field	Visual communication/graphic/brand design	203	38.6
	Industrial/product design	80	15.2
	Product/UI/UX design	120	22.8
	Environmental/interior/landscape design	123	23.4

### 4.2 Measures

All 10 constructs were assessed using four self-report items each. The questionnaire used a five-point Likert scale ranging from 1 = strongly disagree to 5 = strongly agree. Prior research provided the conceptual and measurement basis for the constructs, while the questionnaire items were worded to address students’ use of AIGC tools in design creation. Table 2 summarizes the definition, relevant sources, item numbers, response scale, and contextualization of each construct.

**Table 2:** Construct definitions and measurement overview.

Construct	Definition	Theoretical or measurement basis	Items and design-context wording
Perceived reliability	Dependability of AIGC tools in design work.	Lee and See (2004), Körber (2019)	A1–A4: consistency, accuracy, confidence, and malfunctions.
Perceived ease of use	Perceived effort of learning and using AIGC tools.	Davis (1989)	B1–B4: learning, familiarity, effort, and new functions.
Perceived usefulness	Perceived contribution to design task performance.	Davis (1989)	C1–C4: speed, efficiency, output quality, and practical use.
Perceived intelligence	Perceived ability to respond to design instructions.	Moussawi and Koufaris (2019, 2021)	D1–D4: task completion, interaction, capabilities, and prompts.
Perceived AIGC knowledge	Self-assessed understanding of AIGC tools.	Park (2025), Wang et al. (2025)	E1–E4: familiarity, procedures, peer comparison, and task fit.
AIGC-related anxiety	Tension or concern when using AIGC tools.	Venkatesh (2000), Wilson et al. (2023)	F1–F4: nervousness, ability, control, and use around others.
Self-efficacy	Confidence in one’s ability to use AIGC for design tasks.	Bandura (1997), Compeau and Higgins (1995)	G1–G4: solving problems, independent use, prompts, and output selection.
Trust	Confidence in AIGC tools as support for design work.	Hoff and Bashir (2015), Lee and See (2004)	H1–H4: suggestions, creative value, output quality, and stable support.
Usage intention	Stated willingness to use AIGC tools.	Ajzen (1991), Davis (1989)	I1–I4: future, continued, routine, and recommended use.
Perceived technological dependence	Reported functional reliance on AIGC in design work.	Parasuraman and Riley (1997), Zhang et al. (2025)	J1–J4: reliance, efficiency loss, preference, and wider use.

Each construct was measured with four items on a five-point Likert scale (1 = strongly disagree; 5 = strongly agree). The cited works provide the theoretical or measurement basis; the final item wording refers to AIGC-supported design creation.

Every item in the administered questionnaire referred to AIGC tools rather than technology in general. Most items concerned design creation, while a few addressed broader experiences, such as familiarity with the tools, anxiety when using them in front of others, and routine use. The measures were therefore contextualized to AIGC-supported design work, although not every item was limited to a specific design task.

The definitions of Perceived Reliability, Perceived Ease of Use, and Perceived Usefulness drew on research on trust in automation and the Technology Acceptance Model (Davis, 1989; Hoff and Bashir, 2015; Lee and See, 2004; Venkatesh and Davis, 2000). Perceived Intelligence was conceptualized with reference to research on intelligent agents and AI-enabled systems (Moussawi and Koufaris, 2019, 2021). Perceived AIGC Knowledge was assessed with four self-report items concerning students’ perceived familiarity with AIGC tools, their understanding of how to use the tools in design creation, their understanding relative to peers, and their ability to judge whether a tool suits a design task. The items were informed by discussions of GenAI literacy (Park, 2025), but did not include an objective knowledge test or a performance task.

Trust assessed students’ confidence in AIGC tools and their willingness to rely on generated suggestions in design tasks. Usage intention assessed their stated plans to use the tools in future design work. The four perceived technological dependence items addressed habitual reliance during design creation (J1), an anticipated decline in efficiency without AIGC (J2), preference for AIGC over traditional approaches (J3), and active efforts to involve AIGC in more stages of the creative process (J4). The wording of these items reflects the design context and was informed by research on technology acceptance, automation reliance, AI over-reliance, and GenAI dependence (Glikson and Woolley, 2020; Parasuraman and Riley, 1997; Zhai et al., 2024; Zhang et al., 2025). Higher scores indicate stronger agreement with the corresponding construct.

### 4.3 Data analysis strategy

The data were analyzed using partial least squares structural equation modeling (PLS-SEM) in SmartPLS 4.0. The main aim was to assess the variance explained in several endogenous constructs, particularly Usage Intention and Perceived Technological Dependence, while estimating the proposed direct and specific indirect associations. PLS-SEM was selected for this emphasis on variance explanation and predictive relevance in the context of an extended technology acceptance model. Covariance-based SEM could also estimate these relationships, but assessment of overall covariance fit was not the primary aim of the analysis (Hair et al., 2019; Hair et al., 2022).

First, the reflective measurement model was assessed using outer loadings, Cronbach’s alpha, composite reliability (CR), and average variance extracted (AVE). Discriminant validity was evaluated using the Fornell–Larcker criterion, the heterotrait–monotrait ratio of correlations (HTMT), and cross-loadings (Fornell and Larcker, 1981; Henseler et al., 2015). Potential common method bias was examined with Harman’s single-factor test (Podsakoff et al., 2003) and a supplementary full-collinearity check (Kock, 2015). For the latter, construct scores were reconstructed from standardized item responses and the exported SmartPLS outer weights. Each construct score was regressed on the other nine scores to obtain its full-collinearity VIF; this check was treated as a diagnostic rather than proof that common method bias was absent.

Second, the structural model was assessed using *R*^2^, *Q*^2^, variance inflation factor (VIF), path coefficients, effect sizes, model fit indices, and specific indirect effects. Bootstrapping with 5,000 resamples was used to test the significance of the hypothesized paths and mediation effects. Two-sided 95% percentile bootstrap confidence intervals were reported for the structural paths and specific indirect effects. *R*^2^ values were used to assess the explanatory power of endogenous constructs, while *Q*^2^ was calculated from construct cross-validated redundancy using blindfolding with an omission distance of seven to assess within-sample predictive relevance. Effect sizes were interpreted using Cohen’s (1988) benchmarks, and model fit was reported using SRMR and related supplementary fit indices. Together, these procedures provided a comprehensive assessment of the proposed model.

Because all constructs were measured in a single survey, the path directions represent theoretical expectations, and the indirect effects are statistical estimates under the specified model; neither establishes temporal order or causality.

## 5 Results

### 5.1 Preliminary data screening

Descriptive statistics were examined for all measurement items before the assessment of the measurement model. As shown in Table 3, all items were measured on a five-point Likert scale, with observed values ranging from 1 to 5. The mean scores ranged from 3.46 to 4.09, suggesting that respondents generally reported moderate to relatively high levels of agreement across the constructs. The standard deviations ranged from 0.84 to 1.04, indicating acceptable variation in responses. The absolute skewness values were below 2, and the absolute kurtosis values were below 7 for all items, indicating that the item distributions were within acceptable ranges for subsequent analysis (Kline, 2016).

**Table 3:** Descriptive statistics and normality assessment of measurement items.

Item	Mean	SD	Skewness	Kurtosis
A1	4.09	0.91	−0.96	1.00
A2	3.46	0.94	−0.03	−0.07
A3	3.54	1.04	−0.11	−0.72
A4	3.98	0.91	−0.79	0.71
B1	3.77	0.86	−0.32	0.03
B2	3.85	0.89	−0.70	0.53
B3	3.79	0.97	−0.63	0.11
B4	3.73	0.91	−0.36	−0.02
C1	3.79	0.91	−0.41	−0.15
C2	3.69	0.99	−0.54	−0.16
C3	3.65	0.99	−0.43	−0.31
C4	3.86	0.91	−0.53	0.20
D1	3.85	0.84	−0.43	0.22
D2	3.78	0.91	−0.62	0.25
D3	3.61	0.98	−0.33	−0.54
D4	3.85	0.87	−0.61	0.61
E1	3.87	0.85	−0.50	0.51
E2	3.79	0.87	−0.49	0.23
E3	3.68	0.90	−0.33	0.04
E4	3.55	0.94	−0.33	0.08
F1	3.77	0.90	−0.24	−0.24
F2	3.84	0.85	−0.43	0.11
F3	3.71	0.88	−0.15	−0.31
F4	3.78	0.86	−0.22	−0.08
G1	3.77	0.87	−0.15	−0.43
G2	3.57	0.99	−0.11	−0.69
G3	3.59	0.96	−0.11	−0.60
G4	3.63	0.91	−0.11	−0.31
H1	3.95	0.88	−0.69	0.71
H2	3.75	0.92	−0.51	0.16
H3	3.81	0.90	−0.51	0.17
H4	3.80	0.89	−0.46	0.27
I1	3.95	0.84	−0.53	0.41
I2	3.93	0.85	−0.50	0.21
I3	3.60	0.88	−0.20	−0.04
I4	3.76	0.95	−0.48	−0.18
J1	3.80	0.90	−0.46	0.22
J2	3.62	1.01	−0.38	−0.31
J3	3.54	0.98	−0.19	−0.37
J4	3.78	0.93	−0.51	0.84

A1–A4 = Perceived Reliability; B1–B4 = Perceived Ease of Use; C1–C4 = Perceived Usefulness; D1–D4 = Perceived Intelligence; E1–E4 = Perceived AIGC Knowledge; F1–F4 = AIGC-related Anxiety; G1–G4 = Self-efficacy; H1–H4 = Trust; I1–I4 = Usage Intention; J1–J4 = Perceived Technological Dependence. N = sample size; SD = standard deviation.

### 5.2 Measurement model assessment

#### 5.2.1 Reliability and convergent validity

The reflective measurement model was assessed for indicator reliability, internal consistency reliability, and convergent validity. Because all constructs were specified reflectively, indicator-level VIF was not used as a criterion for evaluating the measurement model (Hair et al., 2022). Following established PLS-SEM guidelines, outer loadings above 0.70, composite reliability (CR) values above 0.70, and average variance extracted (AVE) values above 0.50 were considered acceptable (Hair et al., 2019; Hair et al., 2022). As shown in Table 4, all outer loadings ranged from 0.767 to 0.920, exceeding the recommended threshold. Cronbach’s alpha values ranged from 0.853 to 0.914, CR values ranged from 0.901 to 0.939, and AVE values ranged from 0.696 to 0.795. These results support the reliability and convergent validity of the reflective measures.

**Table 4:** Reliability and convergent validity of the measurement model.

Construct	Items	Loadings	Cronbach’s *α*	CR	AVE
Perceived reliability	A1–A4	0.909, 0.767, 0.870, 0.911	0.888	0.923	0.750
Perceived ease of use	B1–B4	0.884, 0.833, 0.808, 0.824	0.859	0.904	0.702
Perceived usefulness	C1–C4	0.883, 0.847, 0.833, 0.892	0.887	0.922	0.747
Perceived intelligence	D1–D4	0.907, 0.776, 0.841, 0.881	0.874	0.914	0.727
Perceived AIGC knowledge	E1–E4	0.895, 0.850, 0.829, 0.786	0.861	0.906	0.707
AIGC-related anxiety	F1–F4	0.920, 0.864, 0.877, 0.905	0.914	0.939	0.795
Self-efficacy	G1–G4	0.903, 0.811, 0.816, 0.859	0.869	0.911	0.719
Trust	H1–H4	0.898, 0.885, 0.856, 0.860	0.898	0.929	0.765
Usage intention	I1–I4	0.886, 0.844, 0.786, 0.817	0.853	0.901	0.696
Perceived technological dependence	J1–J4	0.898, 0.795, 0.849, 0.877	0.877	0.916	0.732

#### 5.2.2 Discriminant validity

Discriminant validity was assessed using the Fornell–Larcker criterion, the heterotrait–monotrait ratio of correlations (HTMT), and cross-loadings. According to the Fornell–Larcker criterion, the square root of each construct’s AVE should be greater than its correlations with other constructs (Fornell and Larcker, 1981). As shown in Table 5, the square root of the AVE for each construct exceeded its correlations with other constructs, supporting discriminant validity.

**Table 5:** Fornell-Larcker criterion.

Construct	UI	TR	PR	PTD	ARA	PEOU	PI	PU	AK	SE
UI	**0.834**									
TR	0.639	**0.875**								
PR	0.518	0.461	**0.866**							
PTD	0.644	0.423	0.363	**0.856**						
ARA	−0.576	−0.392	−0.362	−0.405	**0.892**					
PEOU	0.458	0.348	0.546	0.317	−0.386	**0.838**				
PI	0.412	0.328	0.397	0.370	−0.469	0.429	**0.852**			
PU	0.432	0.437	0.515	0.314	−0.270	0.532	0.274	**0.864**		
AK	0.667	0.666	0.596	0.457	−0.487	0.465	0.379	0.532	**0.841**	
SE	0.527	0.435	0.349	0.321	−0.569	0.362	0.293	0.302	0.460	**0.848**

Bold diagonal values are the square roots of average variance extracted (AVE); off-diagonal values are correlations between constructs. UI = Usage Intention; TR = Trust; PR = Perceived Reliability; PTD = Perceived Technological Dependence; ARA = AIGC-related Anxiety; PEOU = Perceived Ease of Use; PI = Perceived Intelligence; PU = Perceived Usefulness; AK = Perceived AIGC Knowledge; SE = Self-efficacy.

HTMT was examined as the primary empirical check of discriminant validity (Henseler et al., 2015). For usage intention and perceived technological dependence, HTMT was 0.743, below the prespecified 0.90 criterion. Their latent-variable correlation was 0.644, lower than the square roots of their respective AVEs (0.834 and 0.856; Table 5). The four dependence items also loaded more strongly on their assigned construct (0.795–0.898) than on usage intention (0.511–0.593). These results support separation of the two measured constructs under the reported criteria. They do not, however, eliminate the conceptual proximity introduced by items concerning habitual reliance and preference, which we consider when interpreting the findings.

Perceived AIGC Knowledge and Trust were examined more closely because their structural association was strong. Their HTMT was 0.756 (Table 6), with a 95% percentile bootstrap confidence interval of [0.696, 0.811]; the upper bound was below the prespecified 0.90 criterion. Their latent-variable correlation was 0.666, below the square roots of their respective AVEs (0.841 and 0.875; Table 5). Correlations across the 16 cross-construct item pairs ranged from 0.431 to 0.567, and all items loaded highest on their intended constructs. The knowledge items concerned students’ reported understanding of AIGC tools and their suitability for design tasks, whereas the Trust items concerned reliance on generated suggestions and expectations about output quality and stability. These results support treating the two scales as distinct in this sample.

**Table 6:** HTMT results.

Construct	UI	TR	PR	PTD	ARA	PEOU	PI	PU	AK	SE
UI										
TR	0.730									
PR	0.593	0.513								
PTD	0.743	0.476	0.411							
ARA	0.652	0.432	0.401	0.453						
PEOU	0.533	0.392	0.614	0.361	0.436					
PI	0.474	0.365	0.439	0.420	0.524	0.496				
PU	0.496	0.489	0.574	0.353	0.299	0.608	0.310			
AK	0.779	0.756	0.680	0.525	0.548	0.535	0.433	0.610		
SE	0.611	0.489	0.390	0.368	0.638	0.420	0.332	0.343	0.529	

Values are heterotrait–monotrait ratios of correlations (HTMT); diagonal entries are omitted. Construct abbreviations are defined in the note to Table 5.

### 5.3 Common method bias

Because all constructs were measured in the same questionnaire, we examined potential common method bias using two diagnostic checks. Harman’s single-factor analysis extracted multiple factors, with the first unrotated factor accounting for 36.760% of the item variance (Podsakoff et al., 2003). This result alone cannot exclude common method bias. In the supplementary full-collinearity check, VIFs across the 10 constructs ranged from 1.493 to 3.132; the values for Perceived AIGC Knowledge and Trust were 2.667 and 2.110, respectively. All were below the proposed reference value of 3.3 (Kock, 2015). The checks did not identify severe full collinearity, although shared response tendencies may still have inflated associations among the self-reported constructs (Table 7).

**Table 7:** Harman’s single-factor test.

Test item	Value	Recommended criterion	Interpretation
Total variance explained by extracted factors	70.196%	–	Multiple factors were extracted
Variance explained by the first unrotated factor	36.760%	<40%	Below the stated 40% criterion

Harman’s single-factor test was based on principal component analysis. The result should be interpreted as a diagnostic check rather than definitive evidence that common method bias is absent.

### 5.4 Structural model assessment

#### 5.4.1 Explanatory power and predictive relevance

The explanatory power and predictive relevance of the structural model were assessed using *R*^2^ and *Q*^2^ values. In PLS-SEM, *R*^2^ indicates the proportion of variance explained in each endogenous construct, while *Q*^2^ values greater than zero indicate that the model has predictive relevance for a given endogenous construct (Hair et al., 2019; Hair et al., 2022). As shown in Table 8, the *R*^2^ values of the endogenous constructs ranged from 0.211 to 0.591. Usage intention had the highest explained variance (*R*^2^ = 0.591), followed by trust (*R*^2^ = 0.444), Perceived AIGC knowledge (*R*^2^ = 0.444), perceived technological dependence (*R*^2^ = 0.415), AIGC-related anxiety (*R*^2^ = 0.237), and self-efficacy (*R*^2^ = 0.211). These results indicate that the model explained a moderate proportion of variance in the key endogenous constructs, particularly usage intention and perceived technological dependence.

**Table 8:** Explanatory power and predictive relevance of endogenous constructs.

Endogenous construct	*R* ^2^	Adjusted *R*^2^	*Q* ^2^
Usage intention	0.591	0.588	0.405
Trust	0.444	0.443	0.336
Perceived technological dependence	0.415	0.413	0.300
AIGC-related anxiety	0.237	0.236	0.186
Perceived AIGC knowledge	0.444	0.440	0.306
Self-efficacy	0.211	0.210	0.149

*Q*^2^ represents construct cross-validated redundancy calculated by blindfolding with an omission distance of seven.

Blindfolding with an omission distance of seven yielded positive *Q*^2^ values for all six endogenous constructs (Table 8). The values were 0.405 for Usage Intention, 0.336 for Trust, 0.306 for Perceived AIGC Knowledge, 0.300 for Perceived Technological Dependence, 0.186 for AIGC-related Anxiety, and 0.149 for Self-efficacy. These results support predictive relevance under the blindfolding criterion, but do not establish out-of-sample predictive performance.

#### 5.4.2 Collinearity assessment

Before examining the path coefficients, collinearity among the predictor constructs was assessed using variance inflation factor (VIF) values. In PLS-SEM, VIF values below 5 are generally considered acceptable, indicating that multicollinearity is unlikely to distort the estimation of structural paths (Hair et al., 2019; Hair et al., 2022). As shown in Table 9, all VIF values ranged from 1.000 to 2.044, which were below the recommended threshold. Therefore, multicollinearity was not considered a serious concern in the structural model.

**Table 9:** Collinearity assessment using VIF values.

Predictor	Endogenous construct	VIF
Usage intention	Perceived technological dependence	1.000
Trust	Usage intention	1.870
AIGC-related anxiety	Usage intention	1.634
Perceived AIGC knowledge	Usage intention	2.044
Self-efficacy	Usage intention	1.625
Perceived reliability	Perceived AIGC knowledge	1.654
Perceived ease of use	Perceived AIGC knowledge	1.744
Perceived usefulness	Perceived AIGC knowledge	1.550
Perceived intelligence	Perceived AIGC knowledge	1.285
Perceived AIGC knowledge	Trust	1.000
Perceived AIGC knowledge	AIGC-related anxiety	1.000
Perceived AIGC knowledge	Self-efficacy	1.000

#### 5.4.3 Path coefficients and hypothesis testing

The significance of the structural paths was examined using the bootstrapping procedure with 5,000 resamples. As shown in Tables 10, 11 of the 12 hypothesized paths were supported. Perceived reliability was positively associated with Perceived AIGC knowledge (*β* = 0.371, *p* < 0.001), as were perceived usefulness (*β* = 0.272, *p* < 0.001) and perceived intelligence (*β* = 0.131, *p* = 0.005). However, perceived ease of use was not significantly associated with Perceived AIGC knowledge (*β* = 0.061, *p* = 0.187), indicating that H2 was not supported.

**Table 10:** Standardized path coefficients and 95% bootstrap confidence intervals.

Hypothesis	Path	*β*	STDEV	*t*	*p*	95% CI	Result
H1	Perceived reliability → Perceived AIGC knowledge	0.371	0.046	8.015	<0.001	[0.275, 0.458]	Supported
H2	Perceived ease of use → Perceived AIGC knowledge	0.061	0.047	1.318	0.187	[−0.030, 0.155]	Not supported
H3	Perceived usefulness → Perceived AIGC knowledge	0.272	0.039	7.007	<0.001	[0.194, 0.346]	Supported
H4	Perceived intelligence → Perceived AIGC knowledge	0.131	0.047	2.786	0.005	[0.044, 0.228]	Supported
H5	Perceived AIGC knowledge → AIGC-related anxiety	−0.487	0.033	14.577	<0.001	[−0.550, −0.418]	Supported
H6	Perceived AIGC knowledge → Self-efficacy	0.460	0.037	12.374	<0.001	[0.386, 0.531]	Supported
H7	Perceived AIGC knowledge → Trust	0.666	0.029	22.680	<0.001	[0.606, 0.722]	Supported
H8	Perceived AIGC knowledge → Usage intention	0.296	0.047	6.324	<0.001	[0.199, 0.384]	Supported
H9	AIGC-related anxiety → Usage intention	−0.247	0.037	6.649	<0.001	[−0.318, −0.172]	Supported
H10	Self-efficacy → Usage intention	0.124	0.041	3.042	0.002	[0.046, 0.206]	Supported
H11	Trust → Usage intention	0.291	0.043	6.725	<0.001	[0.206, 0.374]	Supported
H12	Usage intention → Perceived technological dependence	0.644	0.044	14.531	<0.001	[0.554, 0.726]	Supported

**Table 11:** Effect sizes of structural paths.

Structural path	*f* ^2^	Effect size
Usage intention → Perceived technological dependence	0.708	Large
Perceived AIGC knowledge → Trust	0.799	Large
Perceived AIGC knowledge → AIGC-related anxiety	0.311	Medium
Perceived AIGC knowledge → Self-efficacy	0.268	Medium
Perceived reliability → Perceived AIGC knowledge	0.149	Small, approaching medium
Trust → Usage intention	0.111	Small
Perceived AIGC knowledge → Usage intention	0.105	Small
AIGC-related anxiety → Usage intention	0.091	Small
Perceived usefulness → Perceived AIGC knowledge	0.086	Small
Perceived intelligence → Perceived AIGC knowledge	0.024	Small
Self-efficacy → Usage intention	0.023	Small
Perceived ease of use → Perceived AIGC knowledge	0.004	Negligible

Perceived AIGC knowledge was positively associated with trust (*β* = 0.666, *p* < 0.001), self-efficacy (*β* = 0.460, *p* < 0.001), and usage intention (*β* = 0.296, *p* < 0.001), while it was negatively associated with AIGC-related anxiety (*β* = −0.487, *p* < 0.001). In turn, AIGC-related anxiety was negatively associated with usage intention (*β* = −0.247, *p* < 0.001), whereas self-efficacy (*β* = 0.124, *p* = 0.002) and trust (*β* = 0.291, *p* < 0.001) were positively associated with usage intention. Finally, usage intention was positively associated with perceived technological dependence (*β* = 0.644, *p* < 0.001). Overall, the results supported H1 and H3–H12, while H2 was not supported.

#### 5.4.4 Effect sizes

The effect size (*f*^2^) was examined to assess the relative contribution of each predictor to its corresponding endogenous construct. Following Cohen’s (1988) conventional benchmarks, *f*^2^ values of 0.02, 0.15, and 0.35 indicate small, medium, and large effects, respectively. As shown in Table 11, usage intention had a large effect on perceived technological dependence (*f*^2^ = 0.708), and Perceived AIGC knowledge had a large effect on trust (*f*^2^ = 0.799). Trust had only one predictor in the specified model: Perceived AIGC Knowledge. Its *R*^2^ of 0.444 therefore corresponds to *f*^2^ = 0.444/(1–0.444) = 0.799; this value does not arise from competition among multiple predictors of Trust. Perceived AIGC Knowledge also showed medium effects on AIGC-related anxiety (*f*^2^ = 0.311) and self-efficacy (*f*^2^ = 0.268). By contrast, perceived ease of use had a negligible effect on Perceived AIGC knowledge (*f*^2^ = 0.004), which is consistent with the non-significant path coefficient reported above. These findings suggest that the strongest explanatory contributions in the model were associated with Perceived AIGC knowledge and usage intention.

#### 5.4.5 Model fit

Model fit was examined as a supplementary diagnostic. For the saturated model, SRMR was 0.037, below the commonly used 0.08 guideline, whereas NFI was 0.887, slightly below the 0.90 benchmark (Henseler et al., 2014; Hair et al., 2022). There are no universal standalone cutoffs for d_ULS, d_G, or the PLS-SEM chi-square value. In the complete bootstrap results, SRMR, d_ULS, and d_G each exceeded their respective 95% upper reference values (Table 12). Thus, the approximate SRMR criterion was met, but bootstrap-based exact overall fit was not established. These indices are interpreted as supplementary diagnostics alongside the measurement and structural model results.

**Table 12:** Model fit indices of the saturated model.

Fit index	Saturated model	Criterion or reference value	Interpretation
SRMR	0.037	<0.08; bootstrap 95% upper value = 0.033	Below approximate threshold; above bootstrap upper value
d_ULS	1.138	Bootstrap 95% upper value = 0.867	Above bootstrap upper value
d_G	0.532	Bootstrap 95% upper value = 0.498	Above bootstrap upper value
Chi-square	1655.155	No universal standalone cutoff	Reported descriptively
NFI	0.887	> 0.90	Below the benchmark

SRMR = standardized root mean square residual; NFI = normed fit index. The 95% upper reference values were obtained from the complete bootstrap results. Values above these bounds do not support exact overall fit.

Model fit also warrants caution. Although the saturated-model SRMR was 0.037, NFI was 0.887 and bootstrap-based exact fit was not established (Table 12). The reported paths should therefore be interpreted within the specified model, whose overall fit was not consistently supported by these supplementary diagnostics.

#### 5.4.6 Mediation analysis

Specific indirect effects were examined using the bootstrapping procedure with 5,000 resamples. Table 13 presents eight selected indirect paths with one intervening construct. They link the four technological perceptions or Perceived AIGC Knowledge to Usage Intention or Perceived Technological Dependence, which are the outcomes central to this study. The selection was based on the paths examined, not on statistical significance; it includes the non-significant path from Perceived Ease of Use. The model also implies longer paths with multiple intervening constructs, such as Perceived Reliability → Perceived AIGC Knowledge → Trust → Usage Intention, which are outside the scope of this table. Perceived AIGC knowledge was indirectly associated with usage intention through trust (*β* = 0.194, *p* < 0.001), AIGC-related anxiety (*β* = 0.120, *p* < 0.001), and self-efficacy (*β* = 0.057, *p* = 0.005). The direct association between Perceived AIGC Knowledge and Usage Intention remained significant (*β* = 0.296, *p* < 0.001) alongside the three specific indirect effects. Under the specified model, this pattern is consistent with partial statistical mediation through Trust, AIGC-related Anxiety, and Self-efficacy. The other indirect paths in Table 13 are not classified as full mediation because their corresponding direct paths were not estimated.

**Table 13:** Selected specific indirect effects.

Indirect path	*β*	SE	*t*	*p*	95% CI	Result
Perceived AIGC knowledge → Usage intention via trust	0.194	0.031	6.245	<0.001	[0.133, 0.257]	Supported
Perceived AIGC knowledge → Usage intention via AIGC-related anxiety	0.120	0.019	6.509	<0.001	[0.085, 0.157]	Supported
Perceived AIGC knowledge → Usage intention via self-efficacy	0.057	0.020	2.820	0.005	[0.021, 0.100]	Supported
Perceived reliability → Usage intention via Perceived AIGC knowledge	0.110	0.023	4.688	<0.001	[0.065, 0.158]	Supported
Perceived usefulness → Usage intention via Perceived AIGC knowledge	0.081	0.017	4.872	<0.001	[0.050, 0.114]	Supported
Perceived intelligence → Usage intention via Perceived AIGC knowledge	0.039	0.016	2.437	0.015	[0.011, 0.074]	Supported
Perceived ease of use → Usage intention via Perceived AIGC knowledge	0.018	0.014	1.283	0.200	[−0.008, 0.048]	Not supported
Perceived AIGC knowledge → Perceived technological dependence via usage intention	0.191	0.036	5.351	<0.001	[0.121, 0.261]	Supported

SE = standard error; CI = confidence interval. Indirect effects were estimated using bootstrapping with 5,000 resamples. Confidence intervals that do not include zero indicate significant indirect effects.

The results also showed that perceived reliability, perceived usefulness, and perceived intelligence were indirectly associated with usage intention through Perceived AIGC knowledge. By contrast, the indirect effect of perceived ease of use on usage intention through Perceived AIGC knowledge was not significant (*β* = 0.018, *p* = 0.200), which is consistent with the non-significant direct path from perceived ease of use to Perceived AIGC knowledge. Finally, Perceived AIGC knowledge was indirectly associated with perceived technological dependence through usage intention (*β* = 0.191, *p* < 0.001). This estimate describes an indirect association under the specified model. It does not show that changes in Perceived AIGC Knowledge preceded changes in Usage Intention or Perceived Technological Dependence.

Overall, 11 of the 12 hypothesized paths were supported; the path from Perceived Ease of Use to Perceived AIGC Knowledge was not. The specific indirect effects through Trust, AIGC-related Anxiety, and Self-efficacy were significant, as was the indirect effect from Perceived AIGC Knowledge to Perceived Technological Dependence via Usage Intention. These estimates describe associations under the specified cross-sectional model.

## 6 Discussion

### 6.1 Key findings and interpretation

This study examined associations among design students’ perceptions of AIGC tools, Perceived AIGC Knowledge, psychological responses, Usage Intention, and Perceived Technological Dependence. The results were broadly consistent with the hypothesized associations, but they do not establish a transition from adoption to dependence. Perceived Reliability, Perceived Usefulness, and Perceived Intelligence were positively associated with Perceived AIGC Knowledge, with Perceived Reliability showing the largest association among these technological perceptions. The data cannot determine whether these perceptions led students to develop greater understanding of AIGC tools or whether students who felt more knowledgeable evaluated the tools more favorably.

The path from Perceived Ease of Use to Perceived AIGC Knowledge was small and not statistically significant (*β* = 0.061, *p* = 0.187; *f*^2^ = 0.004). Ease of use has an established role in technology acceptance research (Davis, 1989; Venkatesh and Davis, 2000), and related studies have examined effort expectancy in students’ acceptance of digital and generative AI tools (Strzelecki, 2024; Venkatesh et al., 2003; Venkatesh et al., 2012). The outcome examined here, however, was students’ reported understanding of AIGC tools rather than their intention to adopt them. One possibility is that an accessible interface encourages use without necessarily changing that reported understanding. The B1–B4 items also covered learning, familiarity, effort, and new functions. Familiarity may reflect prior exposure to the tools as well as perceived ease of use, which makes this non-significant path harder to interpret. The data cannot establish whether item wording contributed to the result.

Perceived AIGC Knowledge was positively associated with Trust, Self-efficacy, and Usage Intention, and negatively associated with AIGC-related Anxiety. Its association with Trust was particularly strong. Although the two scales met the reported discriminant-validity criteria, students with a generally favorable view of AIGC tools may have rated both their understanding and Trust highly. The study did not measure this broader attitude separately. The responses also do not show whether students who felt knowledgeable had more accurate knowledge or evaluated generated outputs more effectively. GenAI literacy includes abilities that a self-report measure alone cannot establish (Park, 2025).

In the specified model, Perceived AIGC Knowledge had significant indirect effects on Usage Intention through Trust, AIGC-related Anxiety, and Self-efficacy. The indirect effect through Trust was the largest. This pattern is consistent with research linking users’ understanding of automated systems to trust-related judgments (Glikson and Woolley, 2020; Hoff and Bashir, 2015; Lee and See, 2004). The reverse ordering is also plausible: students who trust a tool may judge their own understanding of it more favorably. Because both constructs were measured in the same survey, the analysis cannot determine which ordering better describes their relationship. We did not assess the reliability of the tools used by respondents, however, so the results cannot establish whether their Trust was appropriately calibrated. The associations involving AIGC-related Anxiety and Self-efficacy likewise describe differences in reported feelings and confidence, rather than changes in these states over time (Bandura, 1997; Compeau and Higgins, 1995).

Usage Intention was strongly associated with Perceived Technological Dependence. Students who reported stronger intentions to use AIGC also tended to report greater functional reliance on it in design work. The size of this association does not indicate which came first: intentions could relate to an existing reliance on the tools, and both could reflect aspects of students’ current AIGC experience. Nor does the measure establish that this reliance was harmful.

Perceived AIGC Knowledge also had a significant indirect effect on Perceived Technological Dependence through Usage Intention in the specified model. This result identifies a pattern in the survey responses, not a developmental process observed from knowledge to intention and then to dependence. Longitudinal data would be needed to examine whether the proposed ordering occurs in practice.

### 6.2 Theoretical implications

This study extends technology acceptance research by examining Perceived Technological Dependence alongside Usage Intention. Whereas TAM has primarily addressed perceptions of technology and intention to use it (Davis, 1989; Venkatesh and Davis, 2000), the present model also considers students’ reported functional reliance on AIGC in design work. As discussed in Section 2.2, this reliance is related to continuance intention and habit, but the constructs address different aspects of technology use (Bhattacherjee, 2001; Limayem et al., 2007; Verplanken and Orbell, 2003). Examining intention and reliance together broadens the outcomes considered in AIGC acceptance research; their association does not establish that intention develops into dependence. These findings are grounded in a convenience sample of Chinese design students with prior AIGC experience; whether the same associations appear among first-time users or students in other higher education settings remains to be tested.

The model also gives a more specific role to Perceived AIGC Knowledge. It concerns students’ assessment of their own understanding of AIGC tools, rather than knowledge demonstrated in a test or design task. Its position between technological perceptions and psychological responses makes perceived understanding relevant to the study of AIGC use, while leaving open whether students’ assessments match their actual ability to evaluate generated outputs. This distinction is necessary when interpreting the associations with Trust, AIGC-related Anxiety, and Self-efficacy.

Finally, the separate indirect associations through Trust, AIGC-related Anxiety, and Self-efficacy show the value of considering these responses individually. Research on reliance on automation and on self-efficacy provides a basis for doing so (Lee and See, 2004; Parasuraman and Riley, 1997; Bandura, 1997; Compeau and Higgins, 1995). The findings do not establish whether Trust corresponded to the tools’ actual reliability or whether any of these responses developed after Perceived AIGC Knowledge.

### 6.3 Practical implications

The non-significant path from Perceived Ease of Use to Perceived AIGC Knowledge does not tell us whether students can evaluate generated outputs, which we did not assess. Perceived Reliability had the strongest association with Perceived AIGC Knowledge among technological perceptions, and Trust had the largest indirect association with Usage Intention. In design courses, students could compare outputs for the same brief across prompt revisions, note inconsistent or unsuitable results, and explain why they accepted or rejected each one. We did not test this exercise or whether students’ Trust corresponded to actual tool reliability.

The association between Usage Intention and Perceived Technological Dependence draws attention to how students incorporate AIGC into design work. Because the measure does not distinguish useful reliance from over-reliance, higher scores alone should not be treated as evidence of harm. Course guidelines could specify acceptable AI-supported ideation, disclosure, originality, and responsibility for revising generated material. Sketches, iterations, prompt records, and design rationales could help instructors examine how students reached their final decisions.

For developers of AIGC tools used in design education, side-by-side comparisons, revision histories, and annotation tools could help students examine alternatives and explain their choices. Interfaces could also show how prompt changes affect outputs. The educational effects of these features were not tested in this study.

### 6.4 Limitations and future research

The cross-sectional design limits the interpretation of the proposed model. All constructs were assessed at the same time, so the data cannot establish whether technological perceptions preceded Perceived AIGC Knowledge, whether psychological responses developed afterward, or whether Usage Intention preceded Perceived Technological Dependence. Longitudinal research could test the proposed temporal order, including whether Perceived AIGC Knowledge precedes Trust or whether Trust precedes students’ reported understanding; experimental research could examine these proposed directions under controlled conditions.

All constructs were measured in the same self-report questionnaire at a single time point. This matters particularly for the strong association between Perceived AIGC Knowledge and Trust: students inclined to view AIGC favorably may have rated both measures highly, which could inflate the estimated association. Harman’s single-factor test and the full-collinearity checks in Section 5.3 cannot determine how much, if any, of this association reflects shared response tendencies. One knowledge item also asks students to compare their understanding with that of their peers, so general confidence may affect their answers (Kruger and Dunning, 1999). Future studies could measure Perceived AIGC Knowledge and Trust at separate times, add independently scored knowledge questions, and have assessors evaluate how students select and revise AIGC outputs without seeing their survey responses. This would allow the knowledge–trust association to be examined alongside evidence of what students can actually do.

A further limitation is that actual AIGC use behavior was not directly measured. This study captured students’ perceptions, psychological responses, and usage intention, but did not examine behavioral indicators such as logged frequency of use, time spent with AIGC tools, prompt quality, output revision behavior, or critical evaluation of AI-generated materials. Future research could combine survey data with behavioral logs, design process records, or portfolio-based evidence to examine how perceived dependence is reflected in actual creative practice.

Participants were recruited in China through course-related and student group chats and had prior experience using AIGC tools in design creation. This recruitment approach and single-country sample limit generalizability to other design students, academic fields, and national contexts. The survey did not identify the AIGC platforms respondents used or their familiarity with each. Such differences may be related to their perceptions of the tools and Perceived AIGC Knowledge. Future studies could examine these associations across disciplines and levels of platform experience.

### 6.5 Conclusion

This study examined associations among design students’ perceptions of AIGC tools, Perceived AIGC Knowledge, psychological responses, Usage Intention, and Perceived Technological Dependence. Perceived Reliability, Perceived Usefulness, and Perceived Intelligence were positively associated with Perceived AIGC Knowledge, whereas Perceived Ease of Use was not significantly associated with it.

Students reporting greater Perceived AIGC Knowledge also reported greater Trust, Self-efficacy, and Usage Intention, and lower AIGC-related Anxiety. Trust, AIGC-related Anxiety, and Self-efficacy showed distinct statistical indirect effects in the relationship between Perceived AIGC Knowledge and Usage Intention. Usage Intention was strongly associated with Perceived Technological Dependence, defined here as students’ reported functional reliance on AIGC in design work.

These results extend technology acceptance research by considering perceived reliance alongside intention. They are consistent with the proposed theoretical model, but do not demonstrate that the constructs develop in the order depicted in Figure 1 or establish whether reported reliance is productive or problematic. Future studies can test the proposed sequence over time and compare students’ reported reliance with their design behavior and outcomes.

## Data Availability

The original contributions presented in the study are included in the article/Supplementary material, further inquiries can be directed to the corresponding author.

## Supplementary material

The Supplementary material for this article can be found online at: https://www.frontiersin.org/articles/10.3389/fpsyg.2026.1928967/full#supplementary-material. The English wording of all 40 measurement items is provided in Supplementary material S1.
