# Supplementary material for: Psychological mechanisms of perceived technological dependence on generative artificial intelligence tools: perceived knowledge, trust, anxiety, and self-efficacy
Source: Front Psychol. 2026 Oct 23;17:1928967. doi: 10.3389/fpsyg.2026.1928967 (PMC13739684; doi:10.3389/fpsyg.2026.1928967)
Supplement: Supplementary file 1 [file Supplementary_file_1.docx]

**Supplementary Material S1. Measurement Items**

Item codes and construct names correspond to Table 2 of the manuscript. English wording is translated from the items administered in Chinese. All items used a five-point scale from 1 (strongly disagree) to 5 (strongly agree).

| **Construct** | **Item** | **English wording** |
| --- | --- | --- |
| **Perceived Reliability** | A1 | I am confident about using AIGC tools in design creation. |
|  | A2 | AIGC tools perform consistently when I use them for similar design tasks. |
|  | A3 | AIGC tools can handle design creation tasks correctly. |
|  | A4 | AIGC tools rarely malfunction when I use them for design creation. |
| **Perceived Ease of Use** | B1 | The ease of use of AIGC tools helps me learn to use them more quickly and improve my knowledge of them. |
|  | B2 | I can easily become proficient in using AIGC tools for design creation. |
|  | B3 | I find that using AIGC tools for design creation does not require much mental effort. |
|  | B4 | I can easily learn to use new functions of AIGC tools for design creation. |
| **Perceived Usefulness** | C1 | Using AIGC tools helps me complete design work more quickly. |
|  | C2 | Using AIGC tools for design creation is efficient. |
|  | C3 | AIGC tools improve the quality of my creative work. |
|  | C4 | The usefulness of AIGC tools helps me understand their specific applications in design tasks. |
| **Perceived Intelligence** | D1 | AIGC tools can complete design tasks quickly. |
|  | D2 | AIGC tools can communicate with me in a way I can understand. |
|  | D3 | The intelligent behavior of AIGC tools (their ability to understand and respond) increases my awareness of their capabilities and limitations. |
|  | D4 | AIGC tools can understand my instructions and carry out design creation tasks. |
| **Perceived AIGC Knowledge** | E1 | I have a good understanding of the functions and uses of AIGC tools. |
|  | E2 | I know the main steps for using AIGC tools effectively in design creation. |
|  | E3 | Compared with my peers, I think I understand AIGC tools better. |
|  | E4 | I can tell when AIGC tools are and are not suitable for a design task. |
| **AIGC-related Anxiety** | F1 | I feel nervous when using AIGC tools for design creation. |
|  | F2 | I doubt my ability to use AIGC tools for design creation. |
|  | F3 | I feel unable to control or use AIGC tools. |
|  | F4 | I feel nervous when using AIGC tools in front of other people. |
| **Self-efficacy** | G1 | When using AIGC tools for design creation, I am confident that I can find a solution even if I encounter difficulties. |
|  | G2 | I am confident that I can write prompts to guide AIGC tools in design creation. |
|  | G3 | I can use AIGC tools successfully for design creation without help from others. |
|  | G4 | I am confident that I can select AIGC-generated outputs suitable for my design task. |
| **Trust** | H1 | I am willing to adjust my designs based on suggestions from AIGC tools. |
|  | H2 | I believe AIGC tools can provide ideas that help improve the quality of my design work. |
|  | H3 | I believe the work generated by AIGC tools will be close to what I had in mind. |
|  | H4 | I believe AIGC tools can consistently provide stable, high-quality support for design creation. |
| **Usage Intention** | I1 | I am likely to use AIGC tools for design creation. |
|  | I2 | I intend to continue using AIGC tools in my future design work. |
|  | I3 | I plan to use AIGC tools frequently in my daily life. |
|  | I4 | I would use AIGC tools for design work if recommended. |
| **Perceived Technological Dependence** | J1 | When creating designs, I have become accustomed to relying on AIGC tools to complete most tasks. |
|  | J2 | Without AIGC tools, I would feel that my design work becomes much less efficient. |
|  | J3 | In design creation, I prefer using AIGC tools to traditional methods. |
|  | J4 | I actively seek ways to involve AIGC tools in more stages of my creative work. |
